# Stakeholder experience with artificial intelligence in healthcare: a bibliometric study of satisfaction, trust, acceptance, and patient engagement

**DOI:** 10.3389/fdgth.2026.1842497

**Published:** 2026-06-25

**Authors:** XinKai Li, Chao Jing, ZhiFu Gong, Jing Zhang, Shuai Zhang

**Affiliations:** 1 Department of Human Resources Office, The First Affiliated Hospital of HeBei North University, Jiakou Zhang, China; 2 Department of Industry Conduct Office, The First Affiliated Hospital of HeBei North University, Jiakou Zhang, China; 3 Department of Inspection Office, The First Affiliated Hospital of HeBei North University, Jiakou, Zhang, China; 4 Institute of Traditional Chinese Medicine, HeBei North University, Jiakou Zhang, China; 5 Department of Publicity, The First Affiliated Hospital of HeBei North University, Jiakou Zhang, China

**Keywords:** acceptance, artificial intelligence, bibliometrics, healthcare, patient engagement, satisfaction, stakeholder experience, trust

## Abstract

**Background:**

The implementation of artificial intelligence (AI) in healthcare increasingly depends not only on algorithmic performance but also on how patients and healthcare professionals experience, trust, accept, and engage with AI-enabled systems. Although many bibliometric studies have mapped AI in healthcare, the human-centered evidence base on satisfaction-related stakeholder experience remains fragmented. This study therefore analyzed satisfaction as one component of a broader stakeholder-experience framework that also includes trust, attitude, perception, acceptance, willingness, resistance, and usability.

**Methods:**

English-language articles, reviews, and conference papers published between January 1, 2010 and December 31, 2025 were retrieved from the Web of Science Core Collection and Scopus. After duplicate removal and eligibility screening, 1,794 publications were included. Bibliometric analyses were conducted using Microsoft Excel, VOSviewer, CiteSpace, and bibliometrix.

**Results:**

Publication output increased rapidly after 2020 and reached 696 publications in 2025. The United States and China were the leading contributors, and the Journal of Medical Internet Research was the most productive journal. Keyword and co-citation analyses showed a shift from early work on robotic surgery and clinical decision support toward human-centered topics, including patient satisfaction, trust, attitude, ChatGPT, large language models, explainable AI, ethics, and nursing workforce adaptation. Three interconnected frontiers were identified: large-language-model-mediated clinical communication, explainability and trust in patient-AI-professional relationships, and the educational and professional needs of nurses and other healthcare workers.

**Conclusion:**

This study maps the knowledge structure and thematic evolution of research on stakeholder experience with healthcare AI. The findings show that satisfaction should not be treated as interchangeable with trust, acceptance, or usability; rather, these constructs jointly define a multidimensional human-centered implementation problem. Future research should integrate explainable AI, ethical governance, regulatory compliance, and active patient engagement into the evaluation of healthcare AI.

## 1 Introduction

The integration of artificial intelligence (AI) into clinical diagnosis and treatment has emerged as a pivotal trend in modern medical practice, demonstrating remarkable potential particularly in enhancing diagnostic accuracy, optimizing workflow efficiency, and improving patient experience (1, 2). This research focuses on the satisfaction of both healthcare providers and patients with AI applications, encompassing patients' acceptance of AI-driven tools (e.g., diagnostic models or treatment planning systems) and clinicians' trust in AI-assisted decision-making. The current state of research indicates that both parties generally hold a positive attitude toward AI; in diagnostic scenarios in particular, patients report high levels of satisfaction, and the integration of AI can further improve treatment adherence and clinical outcomes (3, 4). For instance, a multitude of empirical studies have shown that AI-assisted diagnosis can elevate patient satisfaction and facilitate interdisciplinary collaboration (4). Nevertheless, critical issues persist in this field: high research heterogeneity impairs the comparability of study results, and several studies have revealed that the disclosure of AI involvement may slightly reduce satisfaction, necessitating a balanced approach to patient autonomy (1, 5). Additionally, the driving factors of satisfaction remain underexplored in a systematic manner; while AI is widely regarded as a supportive tool rather than a substitute for clinical judgment, ethical concerns and doubts regarding its cost-effectiveness remain unresolved (3, 6). Progress has been achieved in optimizing AI's diagnostic accuracy [e.g., an area under the curve (AUC) ranging from 0.89 to 0.98] and workflow efficiency (e.g., a 35% reduction in reading time) (7, 8). However, the unresolved core challenge lies in the lack of a standardized framework and a comprehensive understanding of the multidimensional factors influencing satisfaction, which hinders the widespread clinical application of AI (1, 3).

A substantial body of empirical research has emerged in this field, aiming to assess the specific impacts of AI on the satisfaction of healthcare providers and patients. Such studies have investigated the acceptance of AI in diagnosis and treatment through questionnaires, clinical trials, and systematic reviews [e.g., employing the Preferred Reporting Items for Systematic Reviews and Meta-Analyses (PRISMA) guidelines]. A systematic review encompassing 45 empirical studies found that 57% of the studies reported moderate to high levels of satisfaction with AI applications, especially in the contexts of treatment adherence and telemedicine (9). With the surge in research efforts, a growing number of articles have been published, leading to the rapid expansion of the relevant literature (10). This explosive growth, however, has made it challenging to capture the current research trends, emerging hotspots, and future directions of the field. Although traditional reviews and meta-analyses can summarize existing evidence (11), they fail to effectively predict dynamic trends or identify the evolution of knowledge structures, as they rely on the static integration of data rather than quantitative network analysis (10). For example, existing reviews have identified a correlation between satisfaction and clinical effectiveness (90% of studies reporting high satisfaction correspond to effective AI applications), yet they have not uncovered the interdisciplinary hotspots or the migration of research frontiers (3). Such limitations highlight the necessity of bibliometric analysis to address the deficiencies of traditional review studies in trend prediction.

A growing body of bibliometric research has already mapped artificial intelligence in healthcare at broad disciplinary or application-specific levels. For example, recent studies have characterized the overall expansion of healthcare AI, the evolution of AI technologies over several decades, and disease- or specialty-specific areas such as retinal disease and autism spectrum disorder (12–15). These studies are valuable for understanding the technological and clinical diffusion of AI **(**Supplementary Table S1**)**. However, they mainly treat AI as a technical, diagnostic, or specialty-specific research domain. They do not systematically map the human-centered literature in which patients, physicians, nurses, and other healthcare professionals evaluate AI through satisfaction, trust, acceptance, attitudes, perceived usefulness, usability, resistance, and engagement. The present study therefore differs from previous bibliometric work in its unit of analysis. Instead of asking only where AI is being applied in healthcare, it asks how AI is experienced, trusted, accepted, or resisted by the stakeholders who must use, explain, supervise, or live with these systems. In this manuscript, satisfaction is not used as a psychometric substitute for trust, acceptance, attitude, or usability. Rather, satisfaction is retained as an outcome-oriented anchor within a broader stakeholder-experience framework, whereas trust, acceptance, perception, usability, willingness, resistance, and concern are treated as related but distinct determinants, mediators, or consequences of healthcare AI implementation. This framing is important because technical accuracy alone does not determine whether AI can be safely and sustainably embedded into clinical workflows or patient-facing services.

## 2 Methods

### 2.1 Data source and search strategy

**Figure 1:**
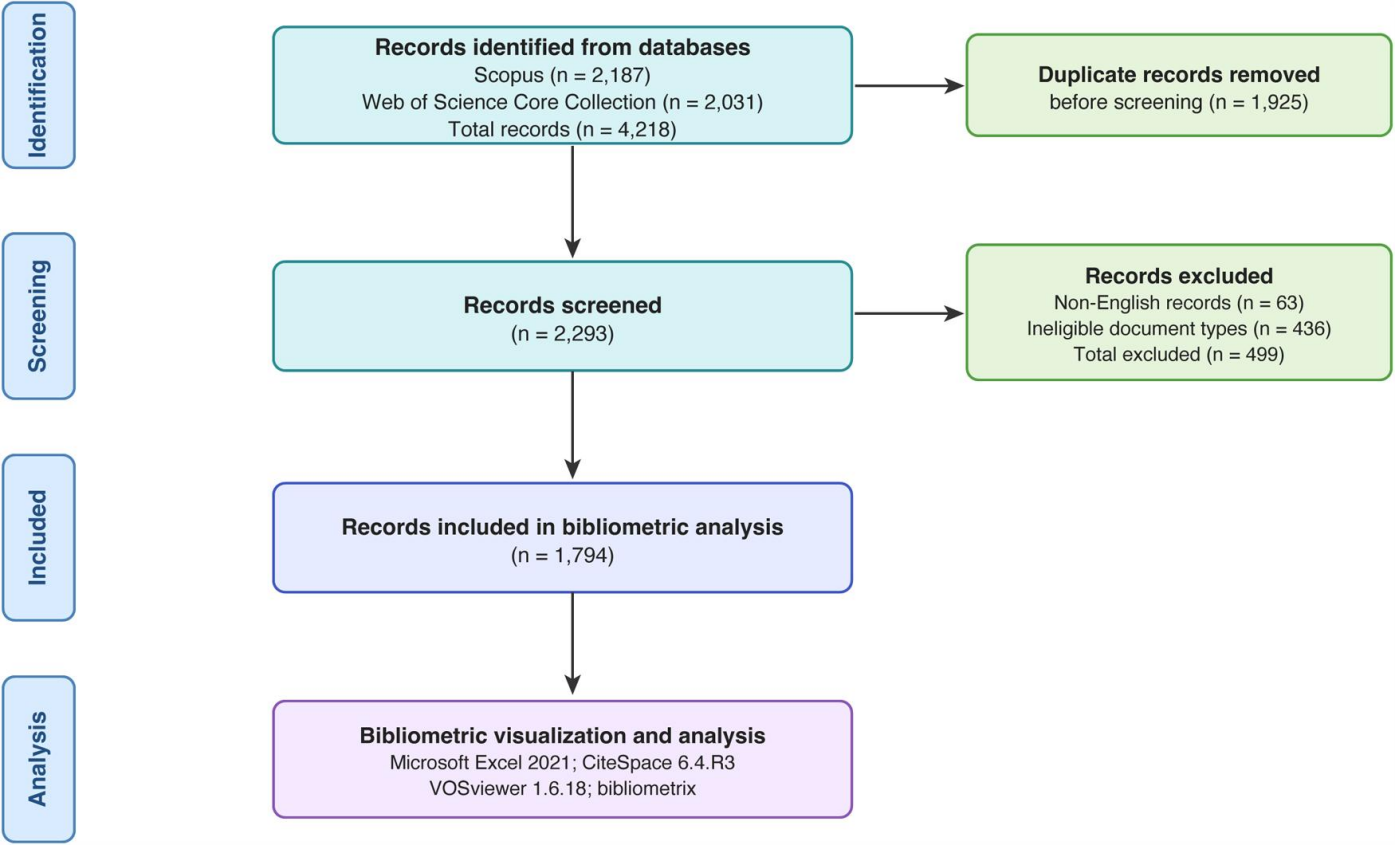
The literature screening process based on scopus and WOSCC illustrates the changes in the number of documents from initial retrieval to final inclusion in the visualization analysis, along with key steps.

Bibliographic records were retrieved from the Web of Science Core Collection (WoSCC) and Scopus (14). The search covered studies published from January 1, 2010, to December 31, 2025. In WoSCC, the query was applied to the title and author keywords fields; in Scopus, the same strategy was applied to the title and author keywords fields. The search combined three groups of terms: healthcare stakeholders (for example, physician, clinician, nurse, patient, caregiver), artificial intelligence-related technologies (for example, artificial intelligence, machine learning, deep learning, natural language processing, large language model, ChatGPT, chatbot, clinical decision support, robotics), and satisfaction- or acceptance-related concepts (for example, satisfaction, acceptance, attitude, perception, trust, preference, usability, adoption, resistance, barriers, and expectations). The initial search retrieved 2,031 records from WoSCC and 2,187 records from Scopus, giving 4,218 records before cleaning. After duplicate removal and eligibility screening (refer to section 2.2 for details), 1,794 English-language publications, including original articles, reviews, and conference papers, were retained for the bibliometric analyses. The records included publication information, subject categories, countries/regions, institutions, authors, journals, references, and keywords (Figure 1).

### 2.2 Multi-database literature integration and deduplication

To construct a unified bibliometric corpus, literature records exported from multiple databases were integrated and deduplicated using a custom R-based preprocessing pipeline. All records were imported in plain-text format and merged into a standardized dataset while preserving the original tagged bibliographic structure. Only valid entries beginning with the publication-type field (PT) were retained for downstream analyses.

Duplicate removal was performed using a hierarchical strategy based primarily on Digital Object Identifier (DOI) matching and secondarily on publication title matching. DOI information (DI field) was first extracted from each record and normalized by converting characters to lowercase and removing redundant whitespace. Records sharing identical normalized DOIs were considered duplicates and collapsed into a single entry. For records lacking DOI information, duplicate detection was subsequently performed using publication titles (TI field). Multi-line titles were reconstructed, normalized to lowercase text, and standardized by compressing inconsistent spacing and formatting. Entries sharing identical normalized titles were regarded as duplicate publications and removed accordingly.

### 2.3 Data analysis

The cleaned dataset was stored as Excel and plain-text files. Publication trends, citation counts, and basic descriptive indicators were summarized in Microsoft Excel 2021. Network construction and visualization were conducted with VOSviewer (version 1.6.18) (16), CiteSpace (version 6.4.R3) (17), and the R package bibliometrix (18). Each tool was used for the part of the analysis for which it is commonly applied: VOSviewer for co-occurrence, co-authorship, and co-citation networks; CiteSpace for burst detection and clustering of cited references; and bibliometrix for descriptive bibliometric summaries and thematic evolution.

The main VOSviewer thresholds were set as follows: keyword co-occurrence analysis, minimum number of occurrences = 10; author co-authorship analysis, minimum number of publications = 3; co-cited author analysis, minimum number of co-citations = 15; co-cited journal analysis, minimum number of co-citations = 75; country collaboration analysis, minimum number of publications = 15; and journal analysis, minimum number of publications = 5. CiteSpace was run with Link Retaining Factor (LRF) = 3.0, Maximum Links Per Node (L/N) = 10, Look Back Years (LBY) = 5, Percentage of Nodes to Label = 1.0%, and Threshold (k) = 25. For cluster interpretation, modularity Q > 0.5 and weighted mean silhouette S > 0.7 were regarded as indicating acceptable clustering quality. These settings were used to reduce noise in the maps while retaining the main knowledge structures relevant to the research question.

### 2.4 Quality-control and bibliometric bias diagnostics

Before final visualization, we performed an additional quality-control and citation-bias diagnostic analysis. Records were screened for retracted publications and publications with expressions of concern using Web of Science and Scopes document-type fields, titles, DOI, PMID, and publication-year information. We also summarized document-type distribution, language distribution, annual output, and citation concentration. Citation concentration was quantified using the Gini coefficient and the citation shares of the top 1%, top 5%, and top 10% most cited records. These analyses were used to evaluate bibliometric visibility bias, not to replace formal clinical risk-of-bias assessment. Because the corpus contained heterogeneous article types, clinical specialties, surveys, reviews, and conference papers, study-level risk of bias and clinical relevance were not scored across all records.

## 3 Results

### 3.1 Publication and citation analysis

**Figure 2:**
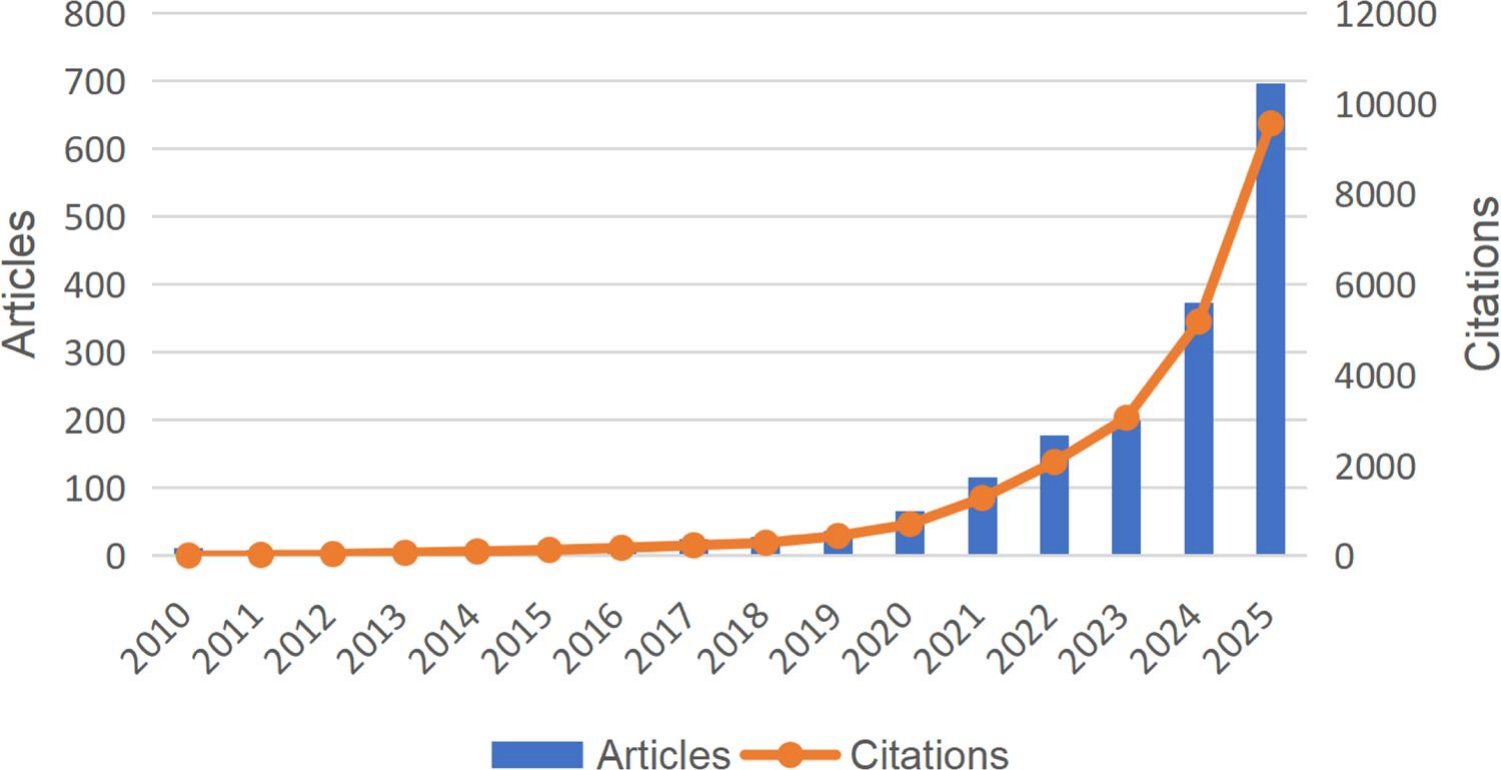
Annual publication volume and citation trends in research literature on patient and physician satisfaction with AI applications in medical diagnosis and treatment from 2010 to 2025.

The publication curve shows a clear change in the pace of research. From 2010 to 2019, studies on satisfaction with artificial intelligence (AI) applications among patients and healthcare professionals appeared only sporadically, and annual citation counts remained low. After 2020, the field entered a faster growth stage. The increase became especially visible between 2023 and 2025, when both publication output and citation frequency rose sharply. In 2025, the number of publications reached 696, representing an 86.6% increase over 2024, and the annual citation count reached 9,554 (Figure 2). Rather than indicating a mature and stable field, this pattern suggests that research interest is still expanding, driven by the rapid clinical diffusion of AI tools and the growing attention paid to acceptance, trust, and user experience.

The sharp rise in 2025 should be interpreted as the result of several converging forces rather than a single bibliometric artifact. First, the post-2022 diffusion of ChatGPT and other large language models created new patient-facing and clinician-facing interaction scenarios, including symptom explanation, medical education, documentation support, and conversational decision support. Second, healthcare AI research increasingly shifted from technical accuracy toward human-centered implementation questions, including trust, transparency, workflow integration, and patient autonomy. Third, regulatory and governance developments, including the EU AI Act and WHO guidance on large multi-modal models for health, increased attention to explainability, accountability, and safe deployment (19, 20). A supplementary diagnostic analysis of the WoS export showed that records containing LLM-, ChatGPT-, or generative-AI-related terms increased from 61 of 347 records in 2024 (17.6%) to 150 of 605 records in 2025 (24.8%). In the same period, records containing ethics-, regulation-, XAI-, trust-, privacy-, consent-, fairness-, or accountability-related terms increased from 111 of 347 records (32.0%) to 248 of 605 records (41.0%). These findings suggest that the 2025 surge reflects both the LLM-driven technology shock and the parallel maturation of ethical and regulatory implementation research.

### 3.2 Distribution of countries/regions

**Figure 3:**
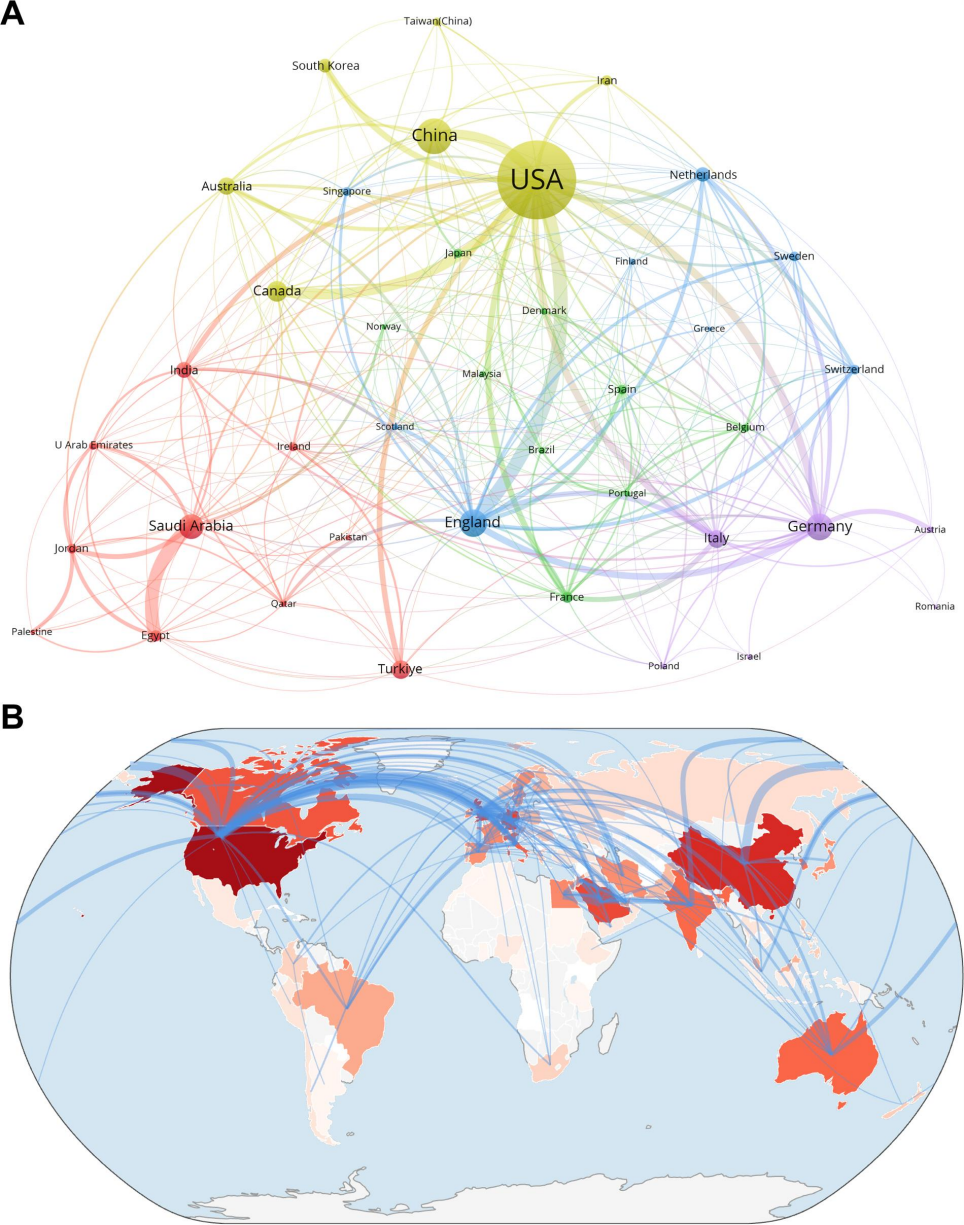
**(A)** national cooperation network visualization: node size represents a country's publication volume, while node color and connecting lines respectively indicate cooperative clustering relationships and association strength. **(B)** Global research collaboration geographic distribution map: color intensity reflects the scale of research output in each country Blue lines represent academic collaboration links between nations.

Country-level output was highly uneven. The United States ranked first, with 568 publications, 9,419 citations, and a total link strength of 214. China ranked second, with 185 publications and 2,523 citations. The United Kingdom, Germany, and Saudi Arabia also contributed substantially (Table 1). The collaboration map adds an important layer to these counts. The United States occupied the central position in the network and maintained frequent links with China, the United Kingdom, Germany, and other active countries. Around this core, several regional groups were visible: a red cluster represented by Saudi Arabia and Turkey, a purple cluster including Germany and Italy, a green cluster around France and Spain, and a blue cluster led by the United Kingdom and the Netherlands (Figure 3A). The geographical heatmap showed the same pattern, with North America, Western Europe, East Asia, and parts of the Middle East forming the main areas of production (Figure 3B). Overall, the field is international, but its collaborative structure is still shaped by a small number of leading countries and regional partnerships.

**Table 1:** Ranking of the top ten major countries/regions in the field of patient and physician satisfaction with AI applications in the diagnostic and treatment process from 2010 to 2025.

Rank	Countries	Documents	Countries	Total Link Strength	Countries	Citations
1	USA	568	USA	9,419	USA	214
2	China	185	China	2,523	England	141
3	England	127	Germany	2,117	Germany	105
4	Germany	124	England	2,079	Italy	76
5	Saudi Arabia	108	Italy	1,391	Saudi Arabia	72
6	Canada	84	Canada	1,296	France	58
7	Turkiye	75	Australia	1,096	China	57
8	Italy	69	Netherlands	1,031	Canada	53
9	Australia	65	France	694	Netherlands	52
10	India	59	Saudi Arabia	686	Egypt	51

### 3.3 Distribution of institutions

**Figure 4:**
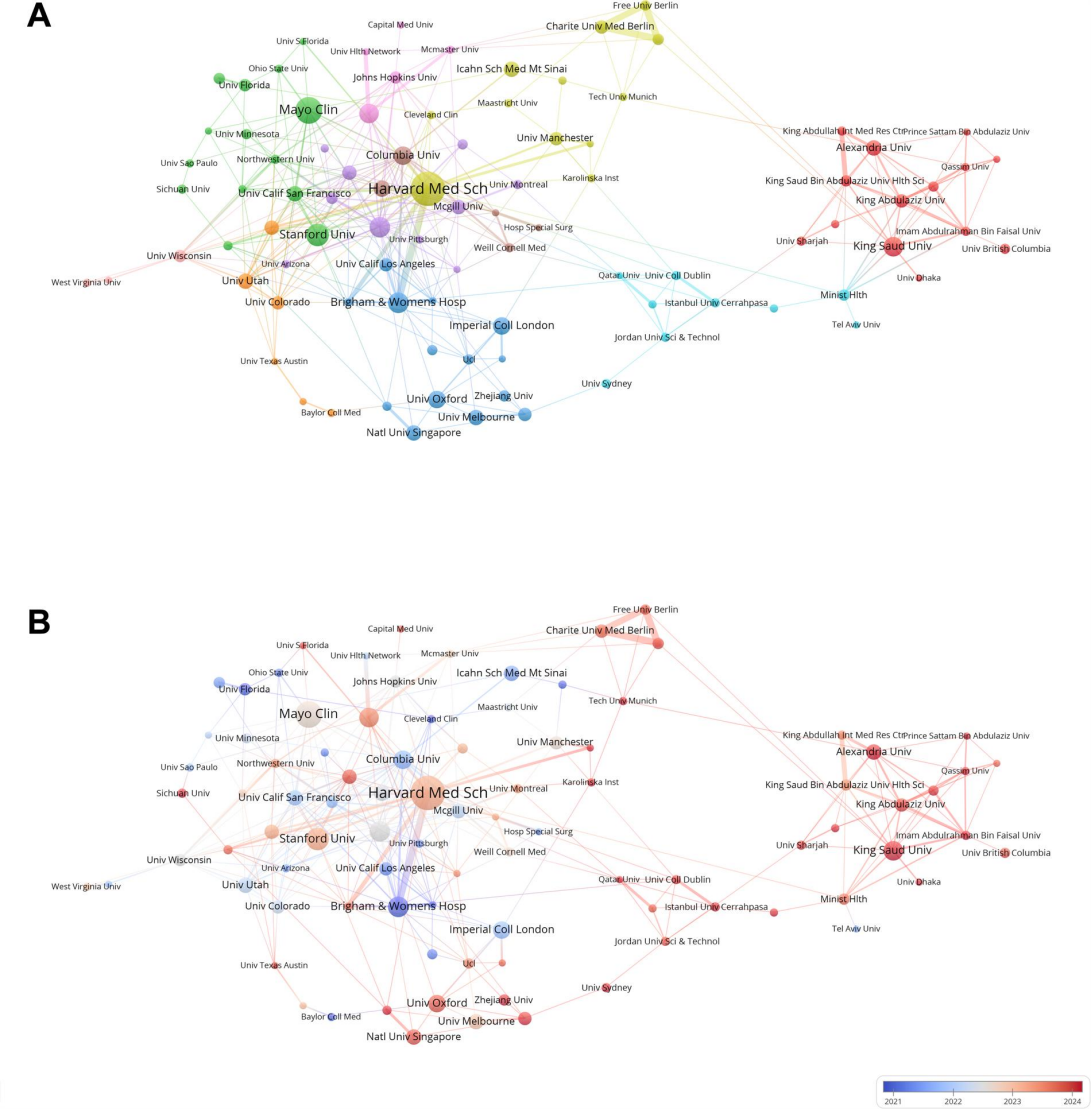
**(A)** institutional cluster analysis. Node colors represent different clusters, node diameters indicate the number of papers published by each institution, and line thickness reflects the degree of collaboration between institutions **(B)** The chart compares each institution's recent contributions in this field with its overall output. The color scale reflects institutional activity levels over the past few years, with red indicating increased influence in this field and blue signifying reduced activity. This highlights institutions that have made significant impacts or experienced declining engagement in this research domain.

Institutional output was also concentrated. Among the top 10 institutions, American institutions accounted for more than half. Harvard Medical School produced the largest number of papers (21), followed by Mayo Clinic (22) and Stanford University (23). Citation impact did not exactly follow publication volume: Brigham and Women's Hospital had 856 citations from 20 papers, while Stanford University and Harvard Medical School also showed high citation counts (Table 2). The collaboration map indicates that these institutions act less as isolated producers than as bridges within larger academic networks. Harvard Medical School, Mayo Clinic, Stanford University, and related institutions formed a dense network on the left side of the map, whereas several Middle Eastern institutions formed a more separate red cluster on the right (Figure 4A). The overlay visualization further suggested that institutions such as King Saud University and University College Dublin have been more active in recent years (Figure 4B). This distribution points to a field in which established biomedical centers provide the main knowledge base, while newer regional groups are beginning to expand their influence.

**Table 2:** Ranking of the top ten major institutions in the field of patient and physician satisfaction with AI applications in the diagnostic and treatment process from 2010 to 2025.

Rank	Institution	Publications	Original Country	Institution	Citations	Original Country
1	Harvard Medical School	33	USA	Brigham and Women's Hospital	856	USA
2	Mayo Clinic	26	USA	Stanford University	655	USA
3	Stanford University	22	USA	Harvard Medical School	615	USA
4	Brigham and Women's Hospital	20	USA	Stevens Institute of Technology	542	USA
5	Duke University	20	USA	Cleveland Clinic	513	USA
6	King Saud University	19	Saudi Arabia	University of California, San Francisco	488	USA
7	University of Toronto	19	Canada	University of Pennsylvania	451	USA
8	Columbia University	18	USA	Imperial College London	419	UK
9	Imperial College London	17	UK	University of Groningen	412	Netherlands
10	University of Oxford	17	UK	Harvard University	409	USA

### 3.4 Distribution of authors and co-cited authors

Author-level analysis showed a developing, rather than settled, research community. Choudhury, Avishek from West Virginia University had the highest number of publications (12) and a high citation count (693). In the co-cited author analysis, Venkatesh, V ranked first, with 187 co-citations and a total link strength of 1,117 (Table 3). The contrast between productive authors and highly co-cited authors suggests that this field draws on two types of knowledge: direct empirical work on AI in healthcare, and broader theoretical literature on technology acceptance, trust, and human-computer interaction.

**Table 3:** Ranking of the top ten major authors in the field of patient and physician satisfaction with AI applications in the diagnostic and treatment process from 2010 to 2025.

Rank	Author	Citations	Total link strength	Countries/regions	Institution	Author	Co- citations	Total link strength	Countries/regions	Institution
1	Choudhury, Avishek	12	693	USA	West Virginia University	Venkatesh, V	187	1,117	USA	Virginia Polytechnic Institute and State University
2	Asan, Onur	8	542	USA	Stevens Institute of Technology	Davis, Fd	141	921	USA	Texas Tech University
3	Akter, Fazila	6	161	Bangladesh	University of Dhaka	Braun, V	139	512	New Zealand	University of Auckland
4	Arvind, Varun	6	49	USA	Columbia University	Blease, C	106	544	Sweden	Uppsala University
5	Bates, David W.	6	86	USA	Harvard Medical School	Rony, Mkk	99	614	Bangladesh	Bangladesh Open University
6	Blease, Charlotte	6	177	Sweden	Uppsala University	Topol, Ej	88	557	USA	Scripps Research Translational Institute
7	Kim, Jun S.	6	49	USA	Icahn School of Medicine at Mount Sinai	Esteva, A	80	534	USA	Artera
8	Lopez, Karen Dunn	6	65	USA	University of Iowa	Choudhury, A	78	442	USA	West Virginia University
9	Mont, Michael A.	6	366	USA	Sinai Hospital of Baltimore	Davenport, Thomas	70	538	USA	New York plastic surgery group
10	Rony, Moustaq Karim Khan	6	161	Bangladesh	Bangladesh Open University	Labrague, Lj	70	460	USA	University of Oklahoma Health Sciences Center

**Figure 5:**
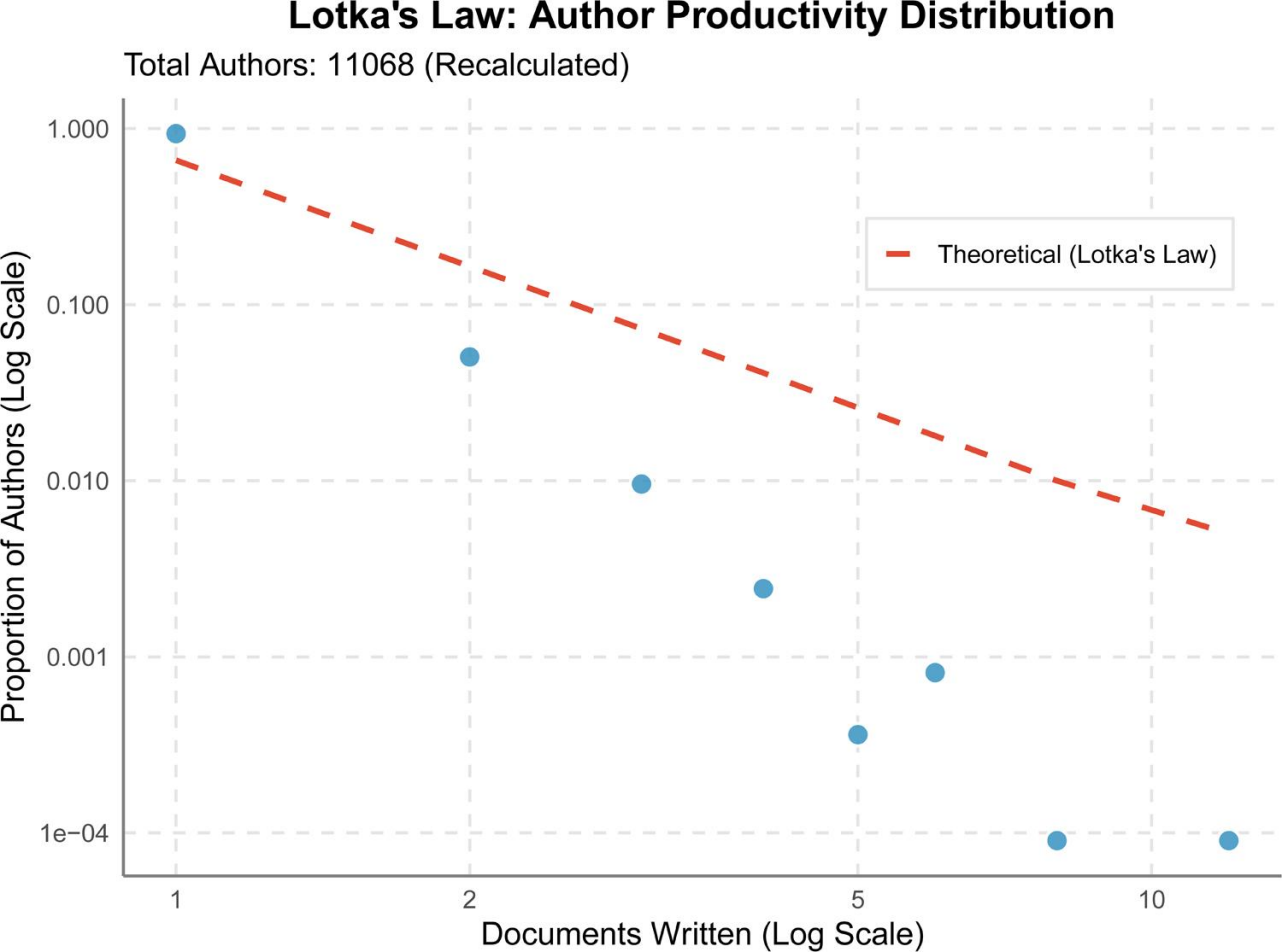
The productivity distribution of authors based on lotka's Law, where blue scatter points represent actual data and the red dashed line indicates the theoretical distribution.

Lotka's Law analysis showed that most authors contributed only one or a small number of papers (Figure 5). The observed distribution generally followed the expected long-tail pattern, but the number of highly productive authors was lower than the theoretical curve. This result is consistent with the recent growth of the topic. Many scholars have entered the field, but a stable core author group has not yet fully formed.

**Figure 6:**
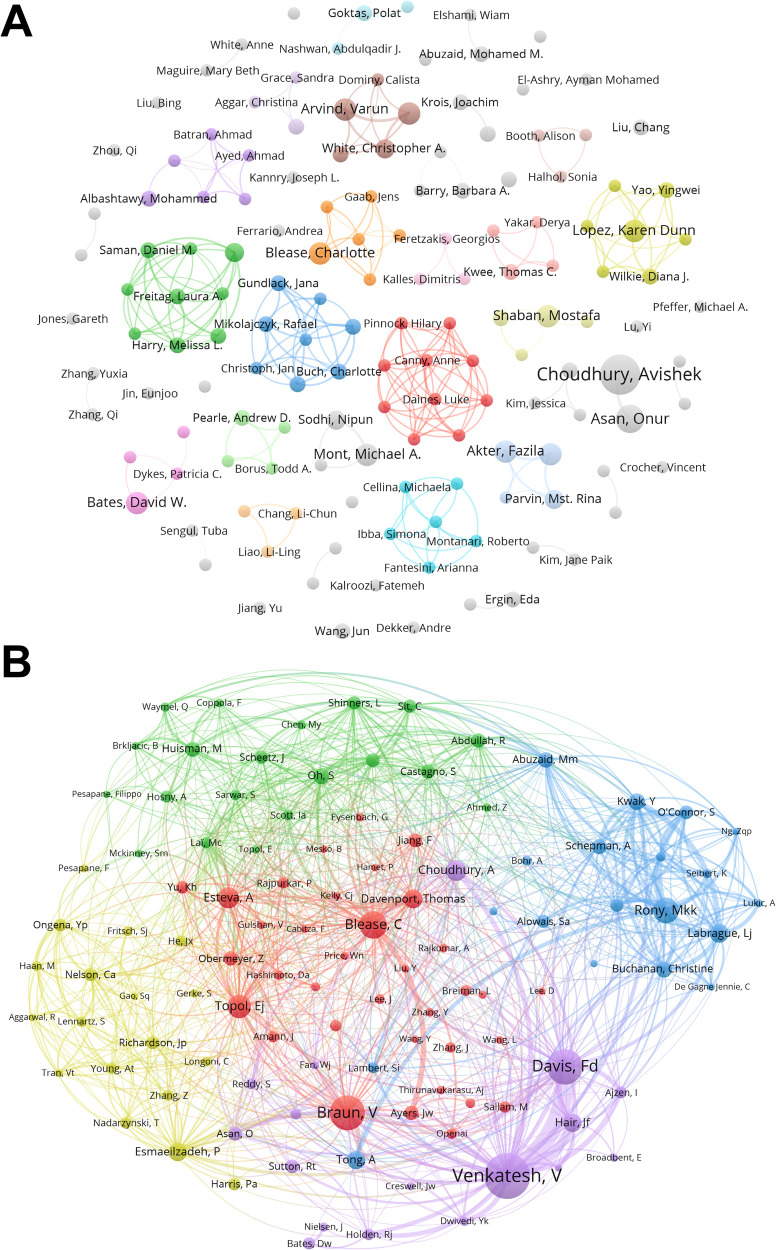
**(A)** this figure displays the author collaboration network within this field, with node colors representing different author groups. **(B)** Co-cited author clustering analysis, where node size indicates co-citation frequency. This visualization, generated using VosViewer, refines and analyzes the association network among co-cited authors in this research domain.

The author collaboration network further supports this interpretation. Several clusters have formed, including groups around Blease, Charlotte; Freitag, Laura A.; and Canny, Anne, while authors such as Choudhury, Avishek and Asan, Onur appear as more independent nodes (Figure 6A). In the co-citation map, the five clusters were not sharply separated from one another (Figure 6B). Their connections indicate that research on satisfaction with AI in healthcare is not built within a single discipline. It brings together medical informatics, clinical communication, nursing, behavioral science, and technology adoption research.

### 3.5 Journal publication analysis

**Figure 7:**
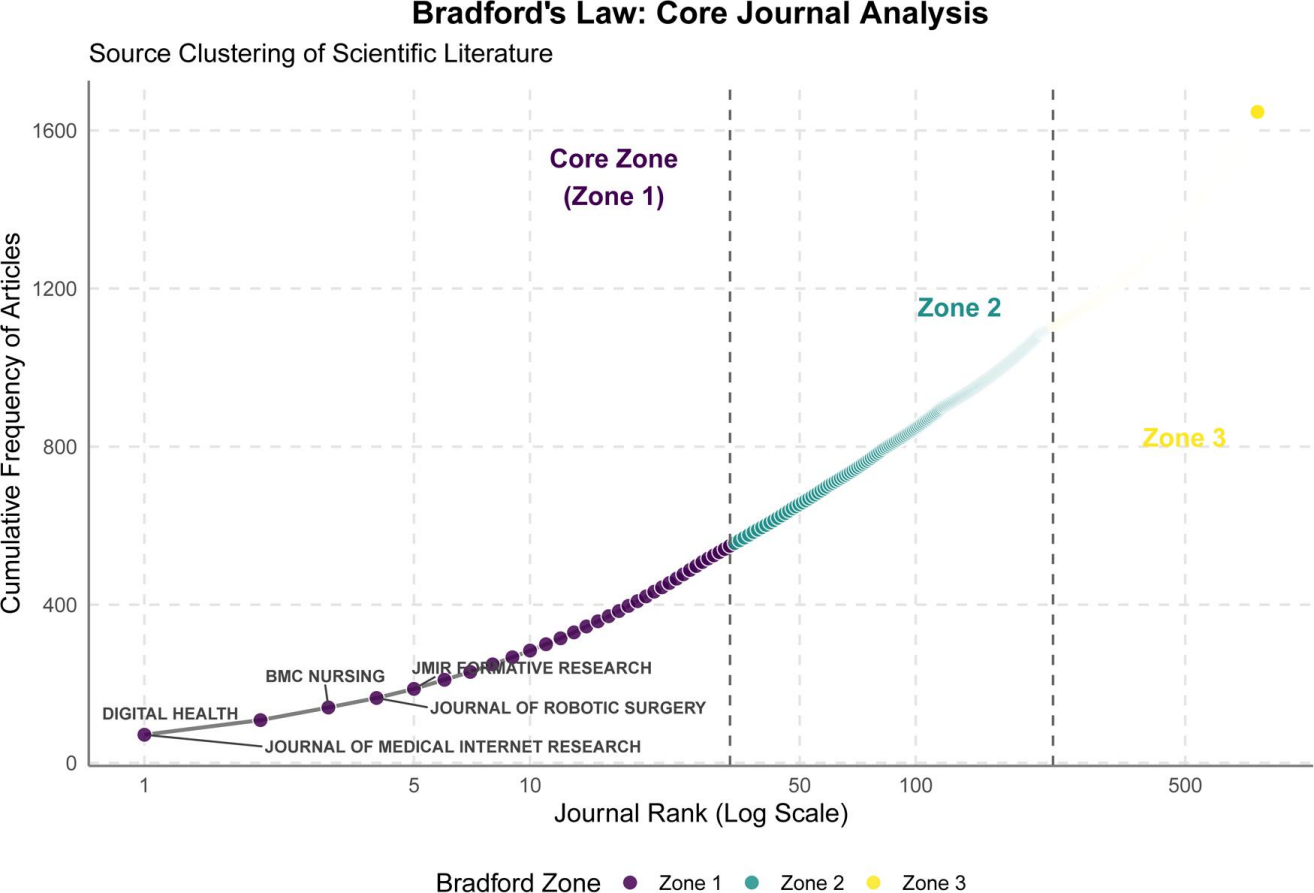
Based on bradford's Law, the core journal analysis map ranks journals by publication volume in descending order and categorizes them into core zone (zone 1), related zone (zone 2), and dispersed zone (zone 3). This visually illustrates the concentrated and dispersed distribution characteristics of literature within journals in this field.

The journal distribution followed the typical Bradford pattern. A small set of core journals carried a large share of the literature, while many journals published only a few related papers. Journal of Medical Internet Research ranked first by publication output, with 76 papers, and was also the most influential co-cited journal, with 1,479 citations and an impact factor of 6.0 (Table 4; Figure 7). Many highly cited journals belonged to Journal Citation Reports (JCR) Q1, indicating that the topic has been taken up by leading journals in medical informatics, digital health, and clinical medicine. At the same time, the wide spread of journals in Bradford Zones 2 and 3 shows that the topic is not confined to one specialty.

**Table 4:** Ranking of the top ten major journals in the field of patient and physician satisfaction with AI applications in the diagnostic and treatment process from 2010 to 2025.

Rank	Journal	Publications	IF(JCR2024)	JCR quartile	Co-Cited-Journal	Citations	IF(JCR2024)	JCR quatile
1	Journal of Medical Internet Research	76	6.0	Q1	Journal of Medical Internet Research	1,479	6.0	Q1
2	Digital Health	37	3.3	Q1	Journal of the American Medical Informatics Association	673	4.6	Q1
3	Bmc Nursing	33	3.9	Q1	JAMA-Journal of the American Medical Association	558	55.0	Q1
4	Journal of Robotic Surgery	25	3.0	Q1	npj Digital Medicine	534	15.1	Q1
5	JMIR Formative Research	24	2.1	Q3	Arxiv	482	N/A	N/A
6	JMIR Human Factors	24	3.0	Q2	International Journal of Medical Informatics	482	4.1	Q1
7	Bmc Health Services Research	20	3.0	Q2	Plos One	482	2.6	Q2
8	Nurse Education in Practice	20	4.0	Q1	BMC Medical Informatics and Decision Making	393	3.8	Q2
9	Healthcare	18	2.7	Q2	Nat Med	383	50.0	Q1
10	Bmc Medical Informatics and Decision Making	17	3.8	Q2	Scientific Reports	341	3.9	Q1

**Figure 8:**
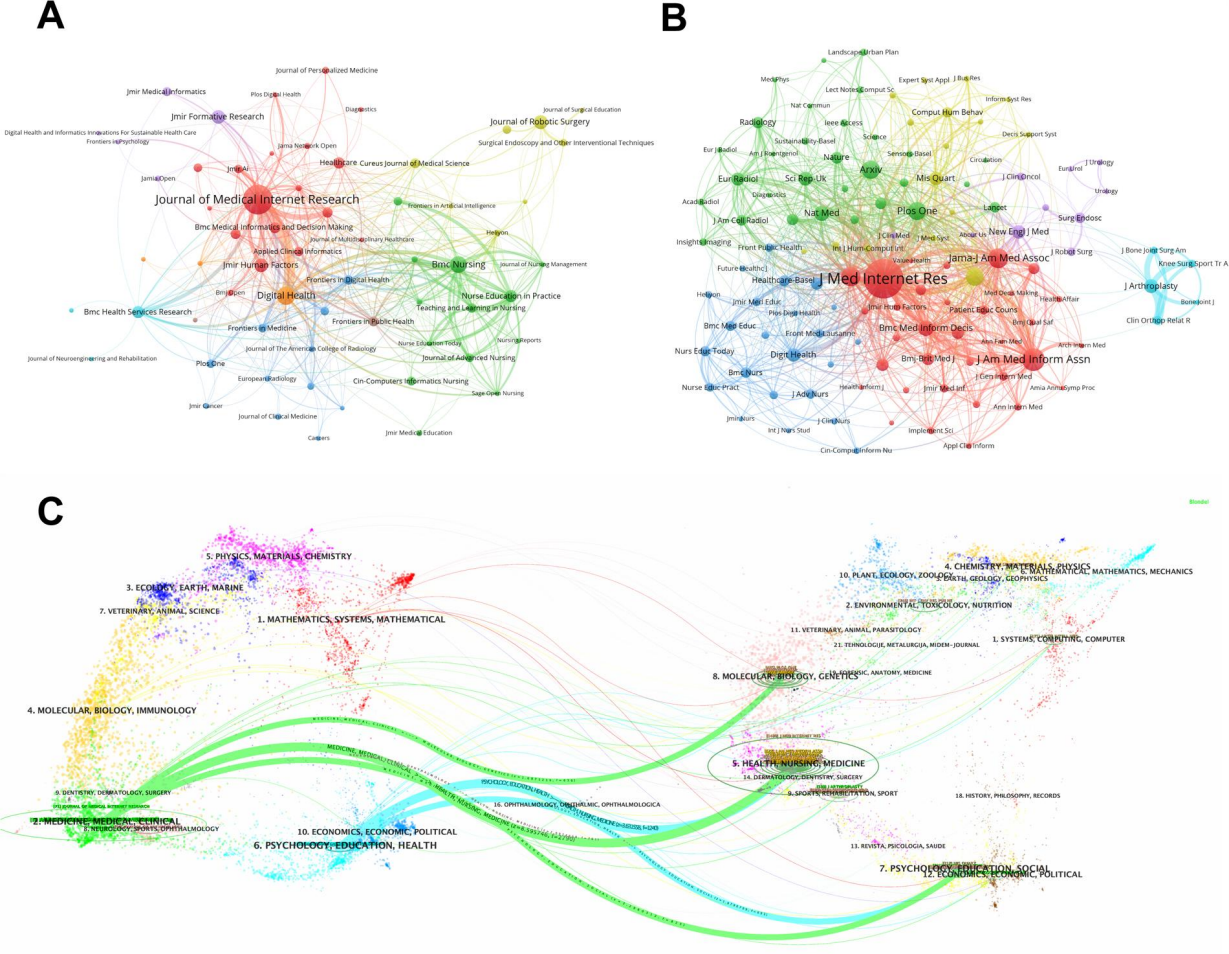
**(A)** visualization and analysis of journal collaboration networks in VOSviewer. Journals within different clusters are distinguished by colored nodes, with node size representing their frequency of occurrence. **(B)** This diagram illustrates co-citation relationships among research journals. Node size reflects co-occurrence frequency, while connecting lines denote co-citation associations. Node dimensions reflect a journal's importance and influence within the network. **(C)** Dual-map visualization of related journals: the left panel shows the citing journal cluster, the right panel displays the cited journal cluster, with colored trajectories between them representing citation relationships.

The journal clusters reveal how the field is organized intellectually. The red cluster, anchored by Journal of Medical Internet Research and BMC Medical Informatics and Decision Making, represents the medical informatics core of the field. The orange and blue clusters connect digital health innovation with broader clinical translation, while the green cluster around BMC Nursing and Nurse Education in Practice reflects the growing presence of nursing education and nursing practice. A separate yellow cluster, including Journal of Robotic Surgery and Surgical Endoscopy and Other Interventional Techniques, captures the earlier surgical and robotic strand of the literature. In the co-citation network, Journal of Medical Internet Research was linked with Journal of the American Medical Informatics Association and JAMA, showing that the most influential knowledge base sits at the intersection of medical informatics, digital medicine, and clinical research (Figures 8A,B). The dual-map overlay also supports this interpretation: journals in medicine, health, nursing, molecular biology, and genetics were frequently connected to cited journals in medicine and clinical research (Figure 8C). Taken together, the journal evidence suggests a shift from technology-specific studies toward a broader user-centered and clinically embedded research agenda.

### 3.6 Keyword analysis

**Figure 9:**
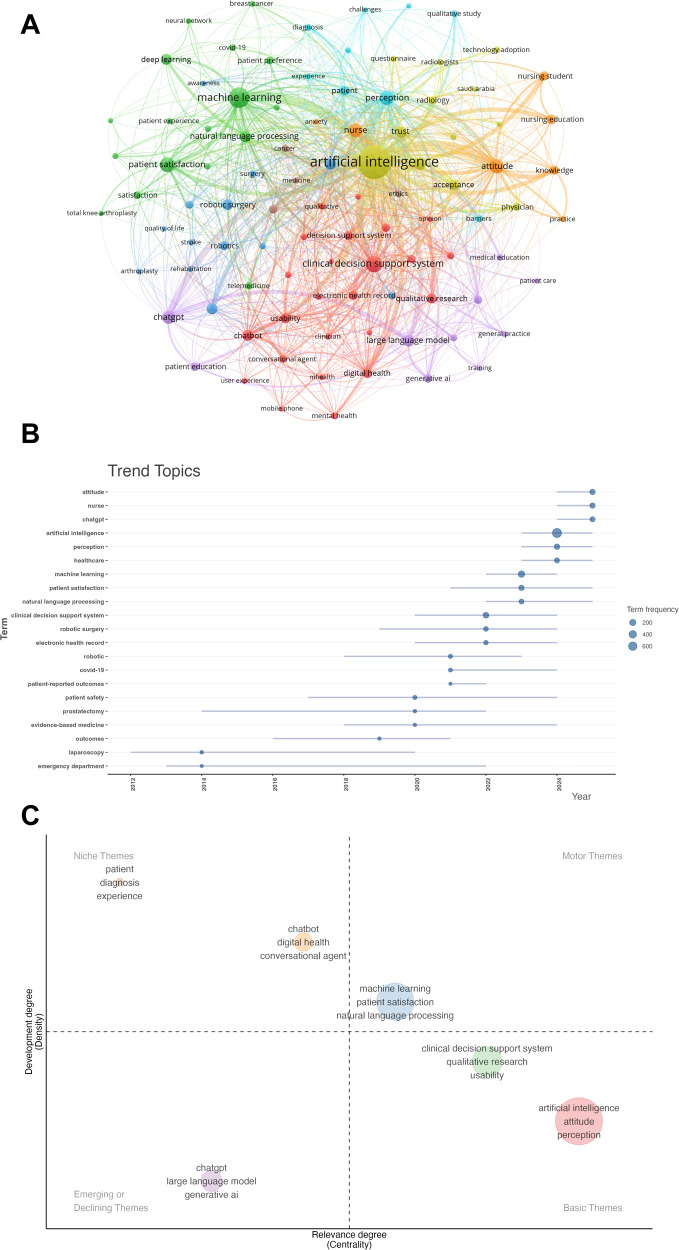
**(A)** keyword co-occurrence network diagram, where node size represents keyword frequency, and color and connections respectively indicate clustering relationships and association strength. **(B)** Trend Topic Timeline Chart: The length of lines and node sizes respectively represent the duration of topic popularity and frequency of occurrence, providing an intuitive visualization of the dynamic evolution of research hotspots in this field. **(C)** Thematic Analysis Diagram for This Field. The horizontal axis and vertical axis represent thematic importance and research density, respectively. The upper-right quadrant represents mature and important core themes; the upper-left quadrant represents highly specialized or technical themes with weak connections to other fields; the lower-left quadrant represents emerging or declining themes; the lower-right quadrant represents themes that are prevalent or auxiliary within this field.

Keyword analysis provided the clearest view of the thematic structure. The most frequent and central term was “artificial intelligence” (726 occurrences; total link strength = 1,600), followed by “machine learning” (225 occurrences; total link strength = 418) and “clinical decision support system” (139 occurrences; total link strength = 286) (Table 5). In the co-occurrence map, these terms did not appear as isolated technical labels. They connected with keywords related to patient satisfaction, attitudes, trust, perception, usability, natural language processing, ChatGPT, large language models, nursing, and robotic surgery (Figure 9A). This pattern indicates that the field has moved beyond evaluating whether AI works technically and has begun to ask how different users experience, accept, and trust AI in clinical settings.

**Table 5:** Ranking of the top twenty major keywords in the field of patient and physician satisfaction with AI applications in the diagnostic and treatment process from 2010 to 2025.

Rank	Keyword	Occurrences	Total link strength	Rank	Keyword	Occurrences	Total link strength
1	artificial intelligence	726	1,600	11	natural language processing	60	165
2	machine learning	225	418	12	survey	60	205
3	clinical decision support system	139	286	13	trust	58	159
4	patient satisfaction	105	181	14	deep learning	55	78
5	attitude	104	358	15	patient perspective	54	114
6	perception	103	350	16	chatbot	53	178
7	nurse	96	224	17	robotic surgery	53	52
8	chatgpt	84	200	18	acceptance	50	174
9	healthcare	76	214	19	qualitative research	44	120
10	large language model	65	177	20	usability	43	133

The clusters in Figure 9A can be read as related thematic communities. The yellow cluster, centered on artificial intelligence, links the broad technical vocabulary of the field with clinical and human-centered terms. The green cluster, around machine learning, connects algorithmic implementation with patient experience and natural language processing. The orange cluster, built around attitude and knowledge, reflects survey- and perception-based studies of patients and healthcare professionals. The red cluster contains clinical decision support systems and electronic health records, showing the importance of embedded AI tools in routine care. The purple cluster, around ChatGPT and large language models, captures the newest conversational AI strand, while the blue cluster reflects the older robotic surgery literature. These clusters should not be interpreted as strict boundaries. Instead, they show how different lines of research overlap around the shared problem of making AI clinically acceptable and trustworthy.

**Figure 10:**
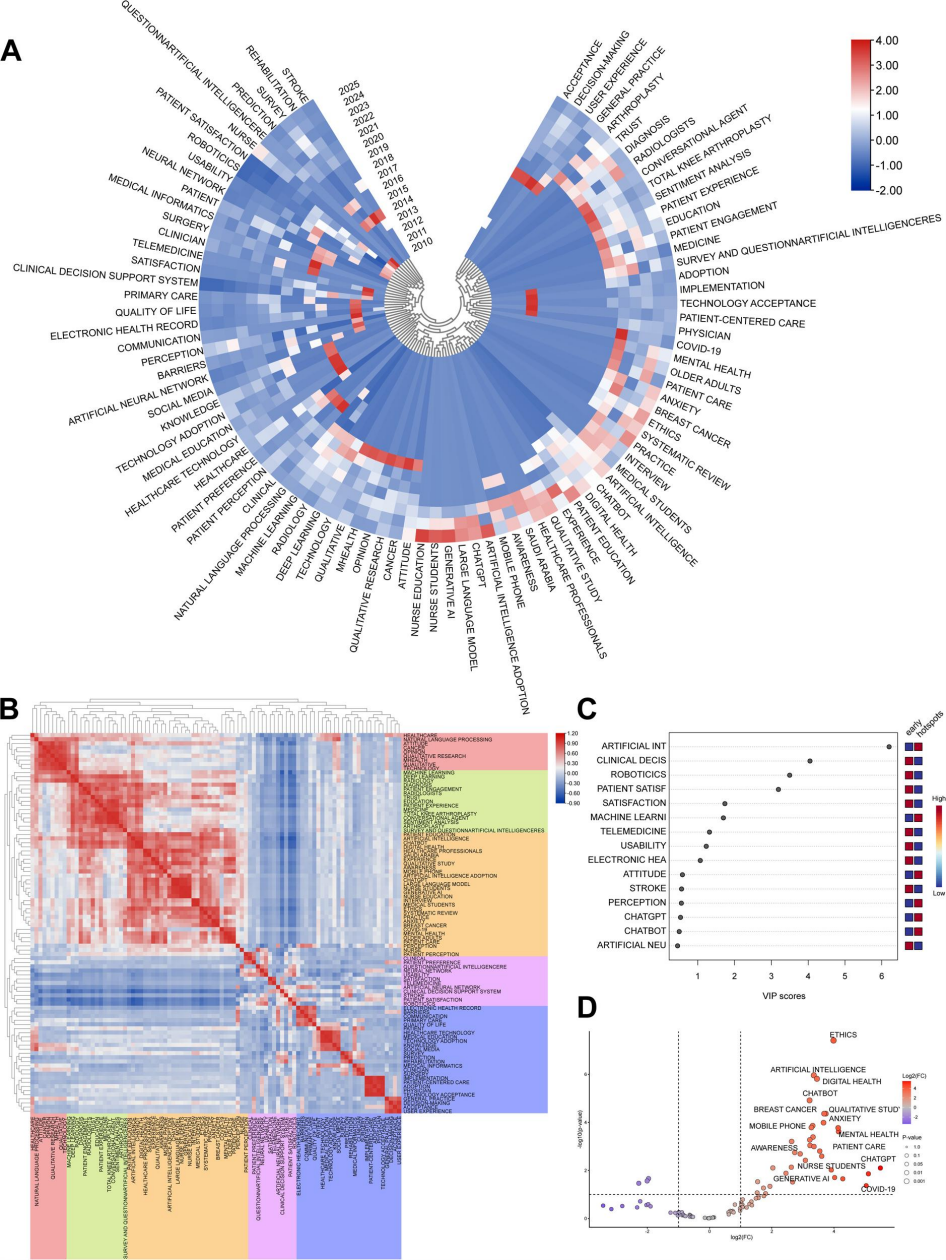
**(A)** the diagram illustrates the correlation of keyword popularity, grouping keywords with similar peak periods into clusters. **(B)** The heatmap displays the prevalence of keywords in related studies, categorizing them into groups based on their popularity during similar time periods and distinguishing them through color coding. **(C)** Variable Importance in Projection (VIP) scatter plot, where higher scores indicate greater explanatory power and importance of the keyword within the model. **(D)** Keyword differential expression volcano plot, where node color and size represent frequency change magnitude and significance, respectively.

The temporal keyword map shows a marked change in emphasis (Figure 9B). Earlier terms, such as prostatectomy, laparoscopy, robotics, and clinical decision support, were mainly linked to procedure-based or system-based applications. After 2019, artificial intelligence and machine learning became more central. Since 2021, ChatGPT, large language model, generative AI, attitude, perception, and nursing-related terms have become more visible. The keyword evolution map and heatmap lead to the same conclusion: the field began with surgical robotics and decision-support tools, then expanded toward patient satisfaction, healthcare professional acceptance, trust, and nursing education (Supplementary Figure S1; Figure 10A).

The four-quadrant thematic map adds another perspective (Figure 9C). Machine learning and patient satisfaction appeared in the motor-themes area, suggesting that they are both well developed and highly connected to the field. Artificial intelligence and clinical decision support system were placed among basic themes, meaning that they provide broad conceptual support across many studies. More specialized or peripheral terms appeared in the other two quadrants, where they may represent either emerging topics or themes with limited connection to the wider network. The keyword popularity analysis was consistent with this pattern. Terms such as artificial intelligence, machine learning, attitude, perception, ChatGPT, and chatbot showed high recent popularity (Figure 10B, C), and the volcano plot highlighted the recent increase in attention to ethics, artificial intelligence, ChatGPT, and large language models (Figure 10D). Overall, the keyword results point to a field that is shifting from AI as a clinical tool to AI as a set of systems embedded in communication, trust, and ethics.

### 3.7 Highly cited reference analysis

Highly cited references show which studies have shaped the field most strongly. The most cited paper was Asan et al.'s 2020 article, “Artificial Intelligence and Human Trust in Healthcare: Focus on Clinicians,” published in Journal of Medical Internet Research (24). This paper is influential because it placed clinicians' trust at the center of AI implementation and discussed reliability, transparency, fairness, and model complexity as key determinants of trust. Other highly cited works by Liu, JL and Shen, LY also received substantial attention (23, 25), indicating that the intellectual base of the field combines trust and acceptance research with technical advances in medical AI (Table 6).

**Table 6:** Ranking of the top fifteen major highly cited references in the field of patient and physician satisfaction with AI applications in the diagnostic and treatment process from 2010 to 2025.

Rank	Author	Article Title	Source Title	Cited	Year	Category	DOI
1	Asan, O; Bayrak, AE et al.	Artificial Intelligence and Human Trust in Healthcare: Focus on Clinicians	Journal of Medical Internet Research	431	2020	Article	10.2196/15154
2	Liu, JL; Wang, CY et al.	Utility of ChatGPT in Clinical Practice	Journal of Medical Internet Research	316	2023	Article	10.2196/48568
3	Shen, LY; Zhao, W et al.	Patient-specific reconstruction of volumetric computed tomography images from a single projection view via deep learning	Nature Biomedical Engineering	237	2019	Article	10.1038/s41551–019-0466-4
4	Palanica, A; Flaschner, P et al.	Physicians' Perceptions of Chatbots in Health Care: Cross-Sectional Web-Based Survey	Journal of Medical Internet Research	224	2019	Article	10.2196/12887
5	Morone, G; Paolucci, S et al.	Robot-assisted gait training for stroke patients: current state of the art and perspectives of robotics	Neuropsychiatric Disease and Treatment	215	2017	Review	10.2147/NDT.S114102
6	Young, AT; Amara, D et al.	Patient and general public attitudes towards clinical artificial intelligence: a mixed methods systematic review	Lancet Digital Health	194	2021	Review	10.1016/S2589-7500 (21)00132-1
7	Horsky, J; Schiff, GD et al.	Interface design principles for usable decision support: A targeted review of best practices for clinical prescribing interventions	Journal of Biomedical Informatics	185	2012	Review	10.1016/j.jbi.2012.09.002
8	Sarwar, S; Dent, A et al.	Physician perspectives on integration of artificial intelligence into diagnostic pathology	NPJ Digital Medicine	184	2019	Article	10.1038/s41746-019-0106-0
9	Blease, C; Kaptchuk, TJ et al.	Artificial Intelligence and the Future of Primary Care: Exploratory Qualitative Study of UK General Practitioners' Views	Journal of Medical Internet Research	177	2019	Article	10.2196/12802
10	Nelson, CA; Pérez-Chada, LM et al.	Patient Perspectives on the Use of Artificial Intelligence for Skin Cancer Screening A Qualitative Study	JAMA Dermatology	172	2020	Article	10.1001/jamadermatol.2019.5014
11	Wang, L; Han, X et al.	Measuring residents' perceptions of city streets to inform better street planning through deep learning and space syntax	ISPRS Journal of Photogrammetry and Remote Sensing	166	2022	Article	10.1016/j.isprsjprs.2022.06.011
12	Abd-Alrazaq, AA; Alajlani, M et al.	Perceptions and Opinions of Patients About Mental Health Chatbots: Scoping Review	Journal of Medical Internet Research	163	2021	Review	10.2196/17828
13	Lambert, SI; Madi, M et al.	An integrative review on the acceptance of artificial intelligence among healthcare professionals in hospitals	NPJ Digital Medicine	162	2023	Review	10.1038/s41746-023-00852-5
14	Jarrassé, N; Proietti, T et al.	Robotic exoskeletons: a perspective for the rehabilitation of arm coordination in stroke patients	Frontiers in Human Neuroscience	148	2014	Review	10.3389/fnhum.2014.00947
15	Marchand, RC; Sodhi, N et al.	Patient Satisfaction Outcomes after Robotic Arm-Assisted Total Knee Arthroplasty: A Short-Term Evaluation	Journal of Knee Surgery	145	2017	Article	10.1055/s-0037-1607450

**Figure 11:**
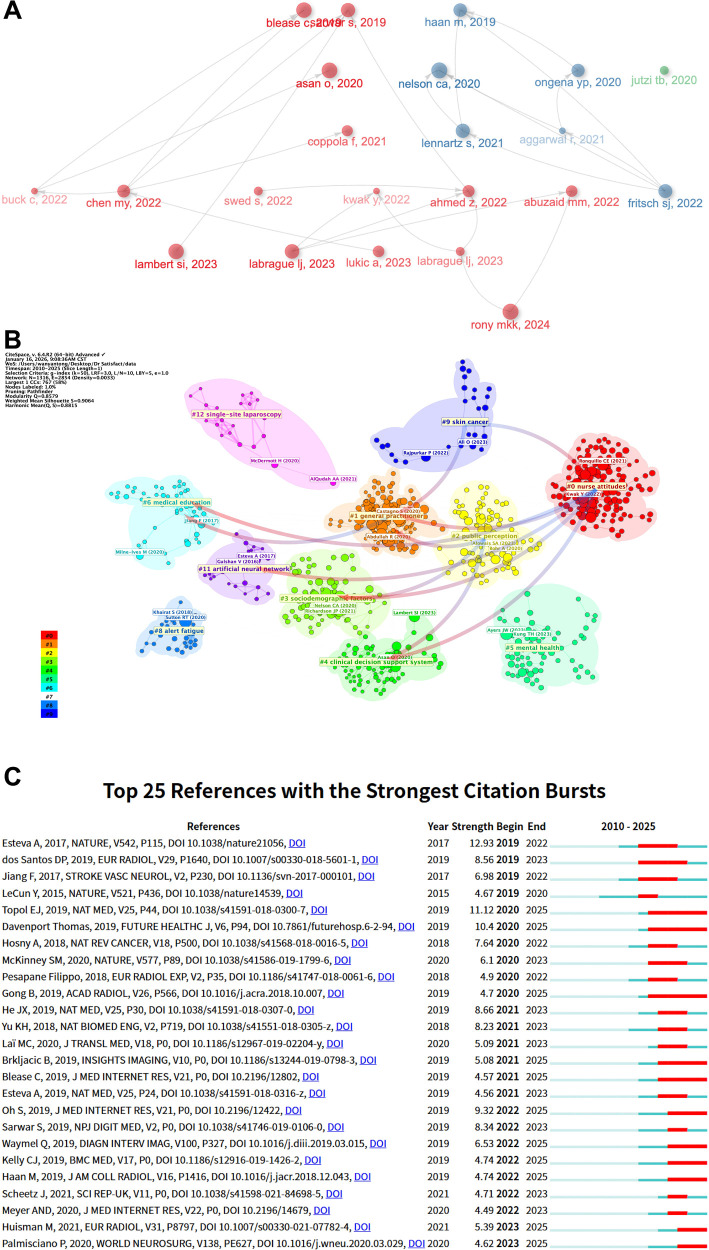
**(A)** the connectivity among the primary 22 citation bursts is demonstrated, depicting the citation interconnections among these articles with arrows. **(B)** References are grouped based on their likeness, where smaller numbers denote larger clusters, with #0 denoting the most substantial cluster. Node size reflects the frequency of co-citations, while the connections between nodes depict co-citation associations. **(C)** The diagram illustrates the 25 primary references characterized by pronounced bursts of citations, denoted by red spikes on the timeline. These spikes signify sudden surges in citation counts, signaling pivotal moments of emerging crucial questions or solutions within the field.

The reference co-citation network helps explain how these influential papers are connected (Figure 11A). Three 2019 qualitative studies by Sarwar et al., Blease et al., and Haan et al. are especially important because they captured the views of physicians, general practitioners, and patients at a time when clinical AI was beginning to move from technical promise to practical implementation (22, 26, 27). These papers helped establish the questions that continue to structure the field: whether clinicians trust AI, how patients understand AI involvement, and how AI changes the clinical relationship. Later works by Asan, Nelson, Labrague, and other teams expanded these questions into broader discussions of human trust, nursing attitudes, and professional adaptation (24, 28, 29).

The cluster visualization in Supplementary Figure 11B shows that the knowledge base is not organized only around AI algorithms. The largest cluster, #0 “nurse attitudes,” indicates that nursing has become a distinct and active line of inquiry rather than a minor extension of physician-centered AI research. Other clusters connect to clinical trust, patient perspectives, medical imaging, and specific AI applications. The citation burst analysis adds a time dimension (Figure 11C). Esteva et al.'s 2017 Nature paper on dermatologist-level skin cancer classification had the strongest burst intensity (12.93), reflecting the early influence of deep learning success in clinical imaging (30). Papers with bursts continuing to the present, including Topol's 2019 article “High-performance medicine: the convergence of human and artificial intelligence” (burst intensity = 11.12), deserve particular attention because they continue to inform current debates about human-AI collaboration, trust, and responsible implementation (31).

Citation-bias sensitivity analysis demonstrated a highly skewed citation distribution within the bibliometric corpus (Supplementary Figure S2). A total of 396 records (23.8%) had zero citations, the citation Gini coefficient was 0.728, and the top 10% of publications accounted for 56.7% of all citations, indicating substantial citation concentration. After quality-control sensitivity filtering, the citation distribution remained highly stable, with a citation Gini coefficient of 0.728 and a top 10% citation share of 56.6%. These findings suggest that although citation visibility is unevenly distributed across publications, the major bibliometric structures and thematic trends identified in this study are robust.

## 4 Discussion

### 4.1 General distribution

The bibliometric analysis results of this study indicated that the research field focusing on the satisfaction of medical staff and patients with the clinical application of artificial intelligence in healthcare is in a stage of vigorous and rapid development. The publication output in this field has exhibited a characteristic of significant and accelerated growth, with an especially prominent upward trend since 2020, which directly reflects the explosive surge in attention paid to this interdisciplinary research direction by both the academic community and clinical practice sectors. From the perspective of geographical distribution characteristics of relevant research, research contributions are highly concentrated in a small number of core regions, among which the United States and China occupy a dominant position in both the number of published papers and academic influence. This distribution pattern is highly consistent with the overall development trends in the fields of artificial intelligence and biomedical research. The characteristics of the international cooperation network revealed that the United States acts as a core node in cross-national collaborations within this field, while the United Kingdom, Germany and Saudi Arabia have also established extensive international cooperation networks, thus presenting an overall research collaboration pattern featuring the coexistence of global interconnection and regional agglomeration. Top research institutions including Harvard Medical School, Mayo Clinic and Stanford University occupy a core hub position in the global cooperation network of this field, which fully embodies their important value as key knowledge producers and nodes for international academic exchanges in this research direction. Analysis of the characteristics of journal publications showed that the *Journal of Medical Internet Research* is the core journal with the highest publication volume and the most remarkable academic influence in this field, followed by other authoritative journals in the fields of medical informatics and digital health. Analysis of highly cited literatures further identified the foundational research achievements in this field, such as the qualitative study conducted by Asan O et al. (24) on clinical physicians' trust in artificial intelligence and a series of fundamental explorations focusing on physicians' perspectives; these achievements together constitute the core knowledge foundation of this research field. These highly cited literatures have not only exerted a profound influence on the research direction of this field, but also established key research topics including trust, acceptance, and human factors in the clinical integration of artificial intelligence as the research core. This thus highlights the necessity of the in-depth integration of technological research and development, clinical practice application and user-centered evaluation, and also reveals the inseparable internal correlations among the three dimensions.

### 4.2 Hotspots and frontiers

Through an in-depth analysis of keyword co-occurrence networks, temporal evolution maps, heatmaps (Figures 9, 10, Supplementary Figure S1), and the clustering of highly cited literature (Figure 11), this study systematically identified the cutting-edge dynamics and core research hotspots in the field of healthcare provider and patient satisfaction with AI applications in clinical diagnosis and treatment. High-frequency keywords including “artificial intelligence,” “attitude,” “ChatGPT,” “clinical decision support,” and “nurse” clearly delineate the core research themes of the current era. Based on these findings and by synthesizing the knowledge structure revealed by bibliometric mapping, the frontier explorations in this field converge on three interrelated research directions.

#### 4.2.1 The evolution of AI technology forms and the shift in research focus toward satisfaction

The role of AI is shifting from a passive tool to an active communicator. Early AI mainly served as an auxiliary diagnostic tool (e.g., imaging analysis systems), with clinicians as its primary interaction objects, aiming to improve decision-making efficiency while involving patients to a low degree (21, 32). However, the emergence of generative AI driven by large language models (LLMs), such as ChatGPT, has enabled it to directly participate in the doctor-patient communication process. By integrating real-time information, providing language translation, and delivering personalized responses, it has played the role of an interactive hub (33, 34). This transformation has given rise to diverse interactive scenarios: in auxiliary communication, AI can real-time analyze dialogues and provide clinicians with feedback on communication skills to optimize interaction quality; in alternative consultation, AI can offer health guidance in scenarios such as chronic disease management, which is highly efficient but requires ensuring that satisfaction is equivalent to that of human consultation (35); ultimately, a new type of Patient-AI medical tools-Physician Interpersonal Trust (PAIP-IT) is taking shape, where AI serves as an information intermediary and coordinator between patients and clinicians (36).

The new interaction model has spawned unprecedented dimensions for satisfaction evaluation. Naturalness of communication has become a key factor, with patients placing high demands on the fluency of AI dialogues and its ability to understand context. Although LLMs are close to humans in semantic coherence, the mechanical nature of their emotional expression still makes 63.3% of patients worry that this will weaken the doctor-patient emotional bond (37). The demand for explanatory credibility has become prominent due to the “black box” problem of AI decision-making; both patients and clinicians require AI to provide diagnostic basis and verify the scientificity of recommendations. As high as 82.9% of patients only accept “AI decisions under clinician supervision”, indicating that transparent disclosure of limitations is the cornerstone of trust (37–40). In addition, expectations for personalization have risen sharply: patients require AI to adjust the complexity of terminology according to their health literacy (33, 41) and integrate personal medical history to generate customized plans (42). However, the current deficiency in emotional recognition and response capabilities has led to approximately 32% of users complaining that the interaction is “lacking in humanization” (43).

The aforementioned changes have directly led to a migration of the overall research focus. The core object of research evaluation has shifted from early clinical outcome indicators (e.g., diagnostic accuracy, efficiency) (32) to in-depth attention to experience indicators, including the quality of doctor-patient communication (44, 45), the effectiveness of emotional support (46), and patients' sense of participation in decision-making. At the same time, studies have revealed many contradictory findings: while AI improves efficiency (e.g., reducing waiting time), it may erode the humanistic care in medical services (47); algorithm standardization ensures fairness but struggles to meet complex individual needs (21); although patients generally recognize the utility of AI (satisfaction rate up to 72%) (3), 83% still insist that human clinicians should occupy a dominant position in diagnosis and treatment (43).

In the future, it is necessary to construct a multi-dimensional satisfaction evaluation system that goes beyond the traditional framework of utility and ease of use, incorporating explanatory transparency (e.g., through algorithm visualization tools), emotional interaction quality (e.g., empathy response index), and personalization adaptability into core indicators (3, 36, 40). At the same time, it is imperative to deepen research on how AI affects the doctor-patient power structure and dynamic relationship, so as to more comprehensively understand and guide this technological transformation.

#### 4.2.2 Deepening research on the dimensions of humanity and trust

Contemporary research has moved beyond preliminary assessments of technical efficacy and usability, entering an in-depth exploration of humanistic and trust-related dimensions. Trust is no longer regarded as a static prerequisite for technology acceptance, but as a dynamically constructed outcome within the triangular relationship of “patient–AI tool–physician”. The PAIP-IT trust framework reveals the multi-agent interactive nature of trust (36). Empirical studies further indicate that AI may erode inherent doctor–patient trust in high-risk scenarios, with a more pronounced impact among female patients (48). The formation of trust follows a dual-path model: on the one hand, AI can indirectly strengthen trust by reducing patient anxiety and improving satisfaction with treatment outcomes (49); on the other hand, algorithmic opacity directly triggers patient concerns over autonomy and privacy, thereby undermining the foundation of trust (50).

Unpacking the “black box” of algorithms is central to establishing trust, and thus technical transparency—known as explainable AI (XAI)—has become a key research focus. Studies demonstrate that providing decision rationales, such as confidence scores and feature visualization, can significantly improve physicians' acceptance of and trust in AI-assisted diagnosis (51, 52), whereas the absence of explanations constitutes a critical barrier to clinical implementation (53, 54). Demand for transparency is multi-dimensional: physicians require understanding of AI logic to fulfill professional responsibilities; patients need explanations commensurate with their health literacy (55); and ethically, XAI serves as a prerequisite for auditing algorithmic fairness and mitigating bias.

The development of trust is strongly moderated by organizational and contextual factors. The depth of integration between AI tools and clinical workflows is critical: if AI functions as an isolated, burdensome add-on, it will provoke physician resistance and markedly reduce trust (56–58). Furthermore, systematic training for physicians and clear accountability mechanisms represent key interventions and institutional safeguards for building organizational-level trust (56, 59, 60). Extensive empirical research has established trust as a core mediating variable in technology adoption. Physicians' adoption intention is not directly determined by technical performance, but almost entirely mediated by trust (61); among patients, trust also acts as a major mediator between AI effectiveness and willingness to use (49). More importantly, trust exhibits a hierarchical transmission effect from “technology → physician → patient”: patients' trust in AI is highly dependent on physicians' endorsement and oversight, and physicians' distrust of AI substantially reduces patient acceptance (43, 62).

The field also confronts intersecting ethical and psychological challenges. Patients commonly experience “dehumanization replacement anxiety”, fearing that AI will weaken the emotional bond between physicians and patients (43, 63); physicians may face fears of deconstructed professional identity (57, 64). Meanwhile, the fairness paradox is prominent: although AI holds promise for reducing subjective bias, inherent biases in training data may entrench or exacerbate health inequities, which requires diverse clinical validation and algorithmic auditing to address (65). Current research has established a four-dimensional analytical framework encompassing technical transparency, psychological acceptance, organizational adaptability, and ethical fairness, marking a fundamental paradigm shift in trust research. Future priorities include developing dynamic trust assessment tools (e.g., empirical validation of the PAIP-IT model) (36), advancing the standardization of XAI (e.g., establishing interpretive guidelines for medical applications) (66, 67), and investigating cross-cultural variations in trust (55, 62). As a central hub for the responsible implementation of AI in healthcare, trust building requires collaborative efforts among technology developers, clinical practitioners, and policymakers.

Unpacking the black box of AI is central to trust, but XAI should be interpreted as more than a technical display of model features. In healthcare, XAI functions as a relational interface among patients, AI systems, and medical professionals. For clinicians, explanations support professional accountability by making AI recommendations auditable, contestable, and easier to integrate with clinical judgment. For patients, explanations must be translated into language that matches health literacy, risk perception, and decision-making needs. For the patient-professional relationship, XAI can either strengthen shared decision-making by clarifying why a recommendation was generated, or weaken trust if explanations appear superficial, inconsistent, or disconnected from clinical reasoning. The recent work integrating chain-of-thought prompting and XAI into a BERT-based diagnostic model illustrates this direction by combining stepwise reasoning logs with attention visualization, LIME, and SHAP to make text-based diagnosis more interpretable to medical professionals (68). However, such explanations require careful validation: a generated reasoning trace should not be assumed to reflect the true causal basis of a model prediction unless it is empirically audited. Therefore, future XAI research should evaluate not only model interpretability but also whether explanations improve physician oversight, patient understanding, informed consent, and calibrated trust.

#### 4.2.3 Independent concerns and educational needs of the nursing workforce

Nurses have emerged as an increasingly prominent and independent focal group in research on physician and patient satisfaction with AI applications in healthcare. This prominence primarily stems from their three core roles in the healthcare system: frontline users, patient educators, and bridges between medical technology and clinical practice. As the group that operates clinical AI systems—including intelligent scheduling tools, clinical decision support systems, and patient monitoring platforms—most directly and frequently, nurses' workflow efficiency and patient outcomes are profoundly affected by AI (69–71). They are beneficiaries of AI-driven improvements, such as optimized human resource allocation (72), yet also the first to confront challenges including tool reliability and ethical dilemmas (71, 73). Accordingly, their satisfaction data serves as a direct indicator for evaluating the practical value of AI (74). Meanwhile, as key providers of patient education, nurses use AI to assist with health instruction and personalized care planning, and their own acceptance and experience with AI strongly shape patients' understanding and trust in the technology (75–77). Furthermore, nurses act as bridges coordinating technology and clinical practice within multidisciplinary teams, and their trust in and willingness to collaborate with AI represent decisive factors in the successful integration and synergistic performance of AI systems (57, 78–80). The interplay of these three roles enables nurses' satisfaction to comprehensively reveal the applicability and challenges of AI at operational, interpersonal, and systemic levels (81–83).

Centered on this focal group, cutting-edge research concentrates on three interrelated themes: AI training, workflow integration, and impacts on professional identity. The demand for targeted AI training is urgent yet largely unmet. Studies consistently reveal weak foundational AI literacy, low practical adoption rates, and a notable knowledge–practice gap among nurses (82). Insufficient training directly contributes to “AI anxiety”—concerns about job replacement or eroded professional value—which in turn suppresses innovative adoption and willingness to use AI (84–86). Emerging research emphasizes the need for personalized, tiered training programs that account for differences in gender, seniority, and experience, while deeply integrating AI ethics, data privacy, and explainability into nursing education and continuing education curricula to foster positive AI beliefs and alleviate anxiety (29, 87–89).

The depth of workflow integration determines both the benefits of AI and nurses' satisfaction. Successful integration—for instance, implementing AI in staffing, documentation automation, or surgical safety verification—effectively reduces administrative burden, improves efficiency, and enhances patient safety, leading to favorable evaluations from nurses (72, 75, 90, 91). However, integration faces substantial barriers: poorly designed AI tools lacking explainability, disrupting workflow autonomy, or raising data privacy concerns can trigger resistance among nurses (73, 76, 92). Consequently, frontier research aims to explore how explainable AI and deeply embedded design can enable seamless, transparent integration of AI recommendations into clinical decision-making, enhancing efficiency while respecting and strengthening nurses' professional judgment (57, 93).

The complex impact of AI on nursing professional identity represents a profound humanistic issue. The introduction of AI may be perceived as an identity threat, challenging the interpersonal care and professional autonomy central to nursing practice, and fostering concerns about emotional disconnection and role redundancy (76, 94). Such perceived threat exacerbates AI anxiety, weakens trust, and indirectly reduces satisfaction and innovative behavior (84, 86). Nevertheless, research also indicates that AI systems designed to augment rather than replace professional competence—such as advanced decision support that reinforces nurses' professional recognition—can consolidate or even elevate their professional identity (78, 79, 86). Thus, emerging research highlights the necessity of proactively addressing and mitigating identity threats in system design and management strategies, while exploring the role of interdisciplinary collaboration in buffering such risks (79, 95).

Independent focus on the nursing workforce signifies a shift toward more nuanced and contextually grounded dimensions in AI-enabled healthcare satisfaction research. Moving forward, customized education to bridge knowledge gaps, human-centered integration to optimize workflows, and identity-sensitive design to preserve professional value represent critical pathways to improving nurses' satisfaction and achieving sustainable, responsible integration of AI in healthcare (29, 57, 72).

### 4.3 Future research directions: regulation, ethics, and active patient engagement

Our bibliometric findings indicate that future work should move from measuring general satisfaction toward evaluating responsible, explainable, and participatory AI implementation. First, ethical and regulatory questions should be integrated into empirical studies rather than discussed only as background concerns. The emergence of keywords related to trust, ethics, ChatGPT, large language models, and XAI suggests that stakeholder experience is increasingly shaped by whether AI systems are transparent, accountable, privacy-preserving, and compatible with professional responsibility. Recent discussion of AI-enabled healthcare services emphasizes that future healthcare transformation depends on governance, service redesign, and responsible implementation rather than technology adoption alone (96). In addition, the EU AI Act introduces legally binding obligations for healthcare-relevant AI systems, and WHO guidance on large multi-modal models highlights risks related to bias, misinformation, privacy, professional oversight, and patient safety (19, 20). Future bibliometric and empirical studies should therefore examine how regulatory frameworks change research priorities, reporting standards, validation practices, and stakeholder trust.

Second, future studies should treat patients as active participants rather than passive recipients of AI-mediated care. Digital transformation can improve information fulfillment by delivering tailored explanations, pre-visit education, symptom tracking, medication guidance, and feedback channels that support patient engagement and safety (97). In AI-enabled care, this means designing systems that help patients ask better questions, understand uncertainty, contribute preferences, and participate in shared decision-making with clinicians. Active engagement should also include patient involvement in AI design, usability testing, consent procedures, and post-deployment monitoring. Such work is especially important for populations with low health literacy, limited digital access, chronic disease burden, or high mistrust of automated decision-making. Future evaluation frameworks should therefore combine satisfaction with patient activation, decisional involvement, explanation comprehension, trust calibration, perceived autonomy, and safety-related outcomes.

### 4.4 Strengths and limitations

This study provides a human-centered bibliometric mapping of healthcare AI research by focusing on stakeholder experience rather than only technical performance or disease-specific AI applications. Compared with previous bibliometric studies of healthcare AI, this analysis specifically connects satisfaction, trust, acceptance, attitude, usability, resistance, explainability, patient engagement, and professional adaptation. This design allows the study to capture the implementation layer of healthcare AI: how patients and medical professionals understand, trust, use, question, or resist AI systems. The use of both WoSCC and Scopus, together with VOSviewer, CiteSpace, bibliometrix, and supplementary quality-control diagnostics, improved the breadth and transparency of the analysis.

Several limitations remain. First, satisfaction, trust, attitude, acceptance, perception, usability, willingness, and resistance are related but distinct constructs. Although they were grouped under a stakeholder-experience search framework to improve retrieval sensitivity, they should not be interpreted as interchangeable psychological outcomes. Second, bibliometric analysis cannot replace systematic review or meta-analytic methods for evaluating clinical effectiveness, methodological quality, or risk of bias. We added basic quality-control filtering for retracted publications and expressions of concern, and we quantified citation concentration as a proxy for bibliometric visibility bias. However, these procedures do not assess study-level risk of bias, selective non-publication of negative findings, or clinical relevance. Third, the analysis remains subject to database coverage, language, indexing, and citation-lag bias. Conference proceedings and computer-science venues may be incompletely represented, while highly cited early studies may exert disproportionate influence on co-citation structures. Fourth, the rapid growth of LLM-related publications in 2025 means that some late publications and citations may not yet be fully stabilized. Future studies should combine bibliometric mapping with systematic review, quality appraisal tools, full-text content analysis, and prospective monitoring of regulatory and implementation outcomes.

## 5 Conclusions

This bibliometric study comprehensively revealed the developmental context, knowledge structure, and research frontiers in the field of physician and patient satisfaction with artificial intelligence applications in clinical diagnosis and treatment through a systematic analysis of 1794 relevant global publications published between 2010 and 2025. The analysis indicated that this field is in an active period of rapid development, with the number of publications accelerating significantly since 2020 and expected to reach a peak around 2030. The distribution of research forces is characterized by both high concentration and global collaboration: the United States occupies an absolute dominant position in terms of output and influence, China is an important contributor, and close regional collaboration networks have been formed in Europe, America, and the Middle East. Top-tier institutions represented by Harvard Medical School, Mayo Clinic, and Stanford University, as well as a cluster of journals centered on the Journal of Medical Internet Research, constitute the key hubs for knowledge production and dissemination in this field.

This study identified and explored in depth three interrelated cutting-edge research hotspots. One such hotspot is the evolution of AI technology forms driving the migration of research focus toward satisfaction: evolving from early auxiliary diagnostic tools to interactive AI represented by large language models, the research focus has shifted from mere evaluation of technical efficacy to in-depth concern for communication quality, explanatory transparency, personalized experience, and the new “Patient-AI-Physician” trust model. Another focus lies in the deepening of research on humanistic and trust dimensions: trust is regarded as the core of dynamic, multi-agent interactive construction, with studies focusing on cracking the “black box” through XAI, exploring the regulatory role of organizational contexts, and confronting interdisciplinary challenges such as ethical fairness and psychological acceptance, thus forming a four-dimensional analytical framework encompassing technology, psychology, organization, and ethics. The third emerging hotspot is the independent attention to the nursing workforce and their educational needs: as key users, educators, and coordinators, nurses' satisfaction, AI knowledge gaps, workflow integration dilemmas, and reflections on professional identity triggered by AI have become new focal points, highlighting the pivotal role of this group in the successful integration of AI.

Despite the fruitful achievements, future research should focus on several directions. It is necessary to construct a multi-dimensional satisfaction evaluation system that goes beyond traditional utility assessment, promote the standardization and clinical application of XAI in the medical field, and develop and validate dynamic trust models (such as PAIP-IT). Efforts should also be made to design personalized, tiered AI competency training programs for healthcare professionals, especially nurses, and more carefully consider the differentiated needs and ethical concerns of different groups (such as those with different cultural backgrounds and professional roles) in system design and policy formulation. In addition, it is crucial to strengthen interdisciplinary and cross-regional collaborative research, particularly cooperation with low- and middle-income countries with high disease burdens, to promote the fairer, more effective, and more human-centered integration of AI in global healthcare. Through bibliometric mapping, this study provides systematic quantitative evidence and directional guidance for grasping the research pattern of this complex field, identifying knowledge gaps, and planning future research paths.

## Data Availability

The original contributions presented in the study are included in the article/Supplementary Material, further inquiries can be directed to the corresponding author.

## Supplementary material

Supplementary Figure S1 2010–2025 evolution chart of top 20 keywords in the field. Horizontal axis: year; Vertical axis: annual keyword ranking.

Supplementary Figure S2 The Lorenz curve illustrates the cumulative distribution of citations across publications included in the study. The pronounced deviation from the diagonal equality line indicates a highly concentrated citation structure, in which a relatively small subset of publications contributes disproportionately to total citation counts. This pattern suggests unequal citation visibility within the field and highlights the importance of interpreting bibliometric influence metrics with caution.
