# Supplementary figures and images for: Stakeholder experience with artificial intelligence in healthcare: a bibliometric study of satisfaction, trust, acceptance, and patient engagement
Source: Front Digit Health. 2026 Jun 25;8:1842497. doi: 10.3389/fdgth.2026.1842497 (PMC13346075; doi:10.3389/fdgth.2026.1842497)

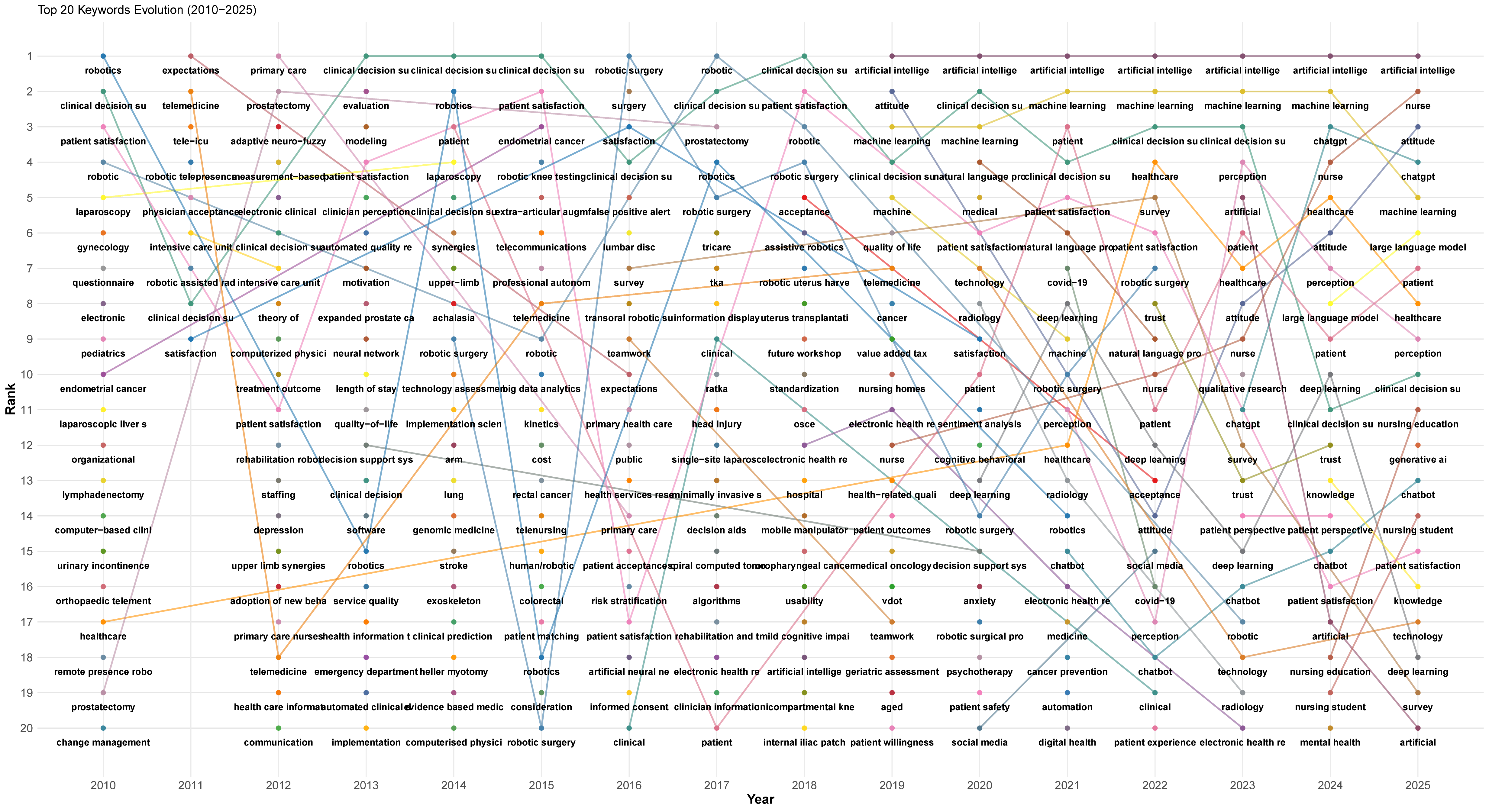

Supplement: Supplementary Figure S1 — 2010–2025 evolution chart of top 20 keywords in the field. Horizontal axis: year; Vertical axis: annual keyword ranking. [file Image1.tif]

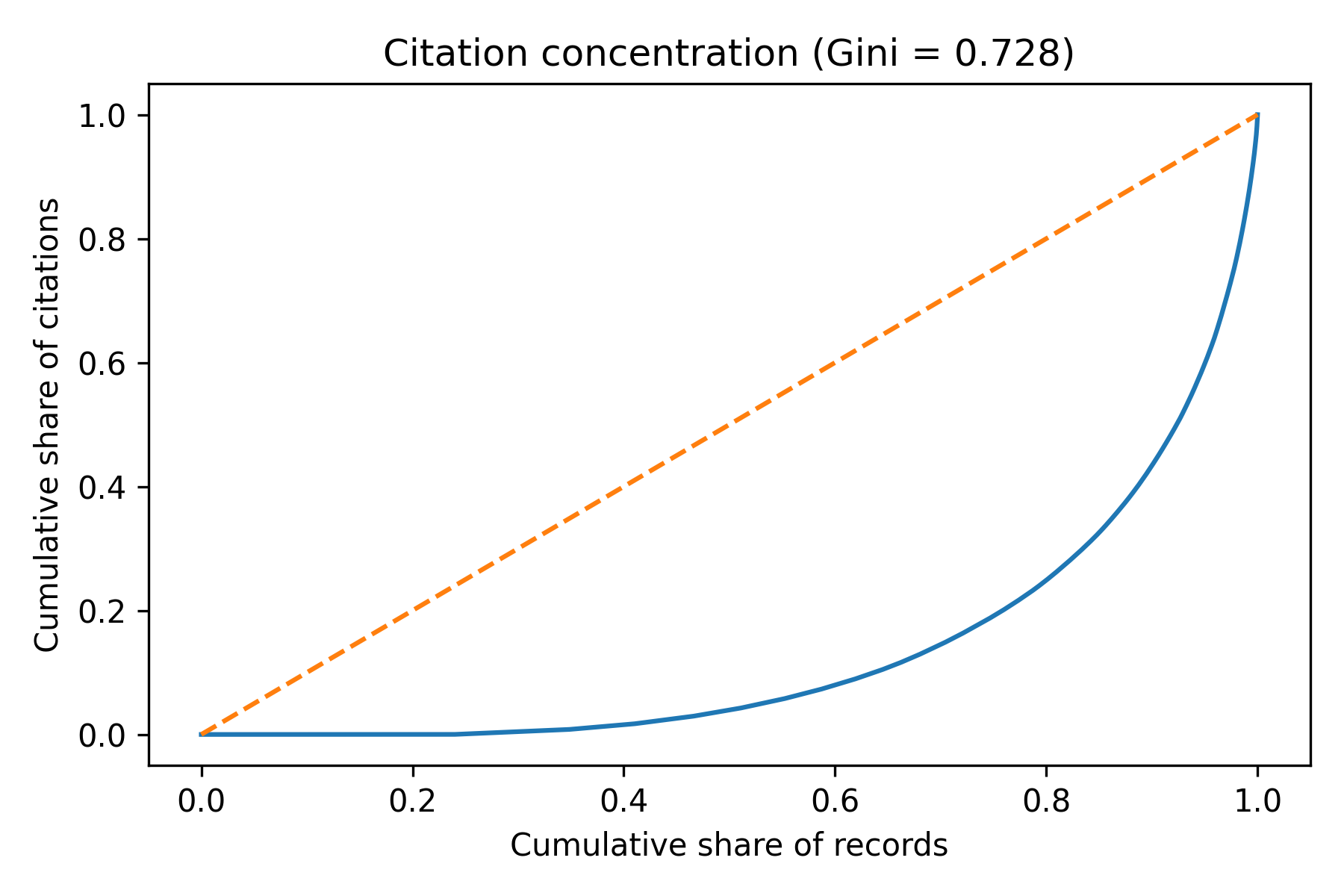

Supplement: Supplementary Figure S2 — The Lorenz curve illustrates the cumulative distribution of citations across publications included in the study. The pronounced deviation from the diagonal equality line indicates a highly concentrated citation structure, in which a relatively small subset of publications contributes disproportionately to total citation counts. This pattern suggests unequal citation visibility within the field and highlights the importance of interpreting bibliometric influence metrics with caution. [file Image2.png]
