# Supplementary material for: Stakeholder experience with artificial intelligence in healthcare: a bibliometric study of satisfaction, trust, acceptance, and patient engagement
Source: Front Digit Health. 2026 Jun 25;8:1842497. doi: 10.3389/fdgth.2026.1842497 (PMC13346075; doi:10.3389/fdgth.2026.1842497)
Supplement: Supplementary file 4 [file Table2.docx]

**Supplementary Table 1. Comparative summary of the present study and previous health-AI bibliometric studies**

| **Study** | **Scope and data source** | **Primary focus** | **Gap relative to the present study** |
| --- | --- | --- | --- |
| Hamsal and Binsar, 2025 | Broad healthcare-service AI landscape; service transformation and future healthcare delivery | AI-enabled healthcare services, operational change, governance, and future service models | Provides broad contextual evolution but does not focus bibliometrically on satisfaction, trust, acceptance, or patient-professional experience. |
| Renganathan Senthil et al., 2024 | Scopus-based bibliometric study of AI in healthcare research | General healthcare AI trends, production patterns, thematic mapping, and emerging technical directions | Maps AI-healthcare research broadly; stakeholder experience and satisfaction-related constructs are not the primary analytical target. |
| Xie et al., 2025 | WoS-based 30-year bibliometric study of healthcare AI | Longitudinal evolution of medical AI, countries, institutions, and broad technical themes including LLMs | Provides macro-level AI-healthcare evolution but does not isolate patient and healthcare-professional satisfaction, trust, or engagement. |
| Present study | WoSCC and Scopus, 2010-2025; stakeholder, AI-technology, and experience-related search terms | Satisfaction, trust, acceptance, attitude, perception, usability, resistance, patient engagement, XAI, and workforce adaptation | Addresses the human-centered implementation layer of healthcare AI and explicitly distinguishes satisfaction from adjacent constructs. |
